# Novel Geometries for Stereotactic Localizers

**DOI:** 10.7759/cureus.15620

**Published:** 2021-06-13

**Authors:** Mark Sedrak, Andres Bruna, Armando L Alaminos-Bouza, Russell A Brown

**Affiliations:** 1 Neurosurgery, Kaiser Permanente Redwood City Medical Center, Redwood City, USA; 2 Medical Physics, Fi.Me. Física Médica SRL, Córdoba, ARG; 3 Medical Physics, Mevis Informática Médica Ltda, São Paulo, BRA; 4 Principal Engineer, Retired, Palo Alto, USA

**Keywords:** stereotactic neurosurgery, stereotactic frame, functional neurosurgery, stereotactic localizers, n-localizer

## Abstract

Introduction: The N-localizer is generally utilized in a 3-panel or, more rarely, a 4-panel system for computing stereotactic positions. However, a stereotactic frame that incorporates a 2-panel (bipanel) N-localizer system with panels affixed to only the left and right sides of the frame offers several advantages: improved ergonomics to attach the panels, reduced claustrophobia for the patient, mitigation of posterior panel contact with imaging systems, and reduced complexity. A bipanel system that comprises two standard N-localizer panels yields only two three-dimensional (3D) coordinates, which are insufficient to solve for the stereotactic matrix without further information. While additional information to determine the stereotactic positions could include scalar distances from Digital Imaging and Communications in Medicine (DICOM) metadata or 3D regression across the imaging volume, both have risks related to noise and error propagation. Therefore, we sought to develop new stereotactic localizers that comprise only lateral fiducials (bipanel) that leave the front and back regions of the patient accessible but that contain enough information to solve for the stereotactic matrix using each image independently.

Methods: To solve the stereotactic matrix, we assumed the need to compute three or more 3D points from a single image. Several localizer options were studied using Monte Carlo simulations to understand the effect of errors on the computed target location. The simulations included millions of possible combinations for computing the stereotactic matrix in the presence of random errors of 1mm magnitude. The matrix then transformed coordinates for a target that was placed 50mm anterior, 50mm posterior, 50mm lateral, or 50mm anterior and 50mm lateral to the centre of the image. Simulated cross-sectional axial images of the novel localizer systems were created and converted into DICOM images representing computed tomography (CT) images.

Results: Three novel models include the M-localizer, F-localizer, and Z-localizer. For each of these localizer systems, optimized results were obtained using an overdetermined system of equations made possible by more than three diagonal bars. In each case, the diagonal bar position was computed using standard N-localizer mathematics. Additionally, the M-localizer allowed adding a computation using the Sturm-Pastyr method. Monte Carlo simulation demonstrated that the Z-localizer provided optimal results.

Conclusion: The three proposed novel models meet our design objectives. Of the three, the Z-localizer produced the least propagation of error. The M-localizer was simpler and had slightly more error than the Z-localizer. The F-localizer produced more error than either the Z-localizer or M-localizer. Further study is needed to determine optimizations using these novel models.

## Introduction

The N-localizer invented in 1978 facilitates frame-based stereotaxis for high-precision neurosurgical targetings, such as for deep brain stimulation (DBS), placement of depth electrodes, stereotactic radiosurgery (SRS), or laser interstitial thermal therapy (LITT) [1,2]. Hence, improvements in frame-based guidance systems are critical for advancements in stereotaxy. Four N-localizer panels affixed to its anterior, posterior, and lateral sides afford the smallest errors. Due to practical considerations, the posterior N-localizer panel is often omitted, resulting in a configuration that comprises three N-localizer panels [3]. In order to promote patient comfort, minimize claustrophobia, and simplify the design, the removal of the anterior and posterior N-localizer panels creates a configuration that comprises only two N-localizer panels, wherein one N-localizer panel is affixed to each lateral side of a rectangular frame [4]. However, using classic N-localizer mathematics, two N-localizer panels are insufficient to determine the spatial orientation of a single computed tomography (CT) or magnetic resonance imaging (MRI) image plane in a three-dimensional (3D) space. In an attempt to rectify this defect of only two N-localizer panels, extrapolation across multiple CT images can be performed, or the nominal scalar dimensions of the image can be used. However, each technique assumes prior or relatable information and is prone to extra, uncontrolled errors. Moreover, extrapolation across multiple CT images violates the guiding principle that provided an impetus for the invention of the N-localizer, which is that each CT image lies in its coordinate system, independent of all other CT images [5].

Better solutions that affix novel localizer panels to only the two lateral sides of a frame are the report's focus. These configurations provide sufficient information to determine the spatial orientation of a single CT or MRI image plane in 3D space. We propose three novel localizer systems and discuss Monte Carlo (MC) simulations that test their viability and potential to propagate errors. We call these localizers the M-localizer, F-localizer, and Z-localizer.

## Technical report

Frame-based Stereotactic Localization

**Figure 1:**
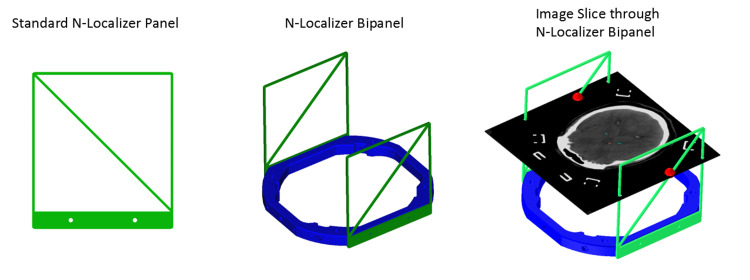
N-localizer panel (left), bipanel (middle) and of an axial computed tomography slice within localizer volume (right). An axial image slice through the localizer system yields only two 3D points (red), which are insufficient for stereotactic localization of the image.

The mathematics for frame-based stereotactic localization has been studied extensively. In particular, the method for computing the stereotactic matrix includes three or more 3D points per image slice. This stereotactic matrix converts 3D coordinates to two-dimensional (2D) coordinates or 2D to 3D. It has been shown previously that the use of an overdetermined system of equations affords an incremental minimization of error at a given target [3]. However, with the introduction of a 2-panel (bipanel) localizer system, the ability to localize a single image yields insufficient information because only two of the three required 3D points are present (Figure 1).

Novel Localizer Bipanels

**Figure 2:**
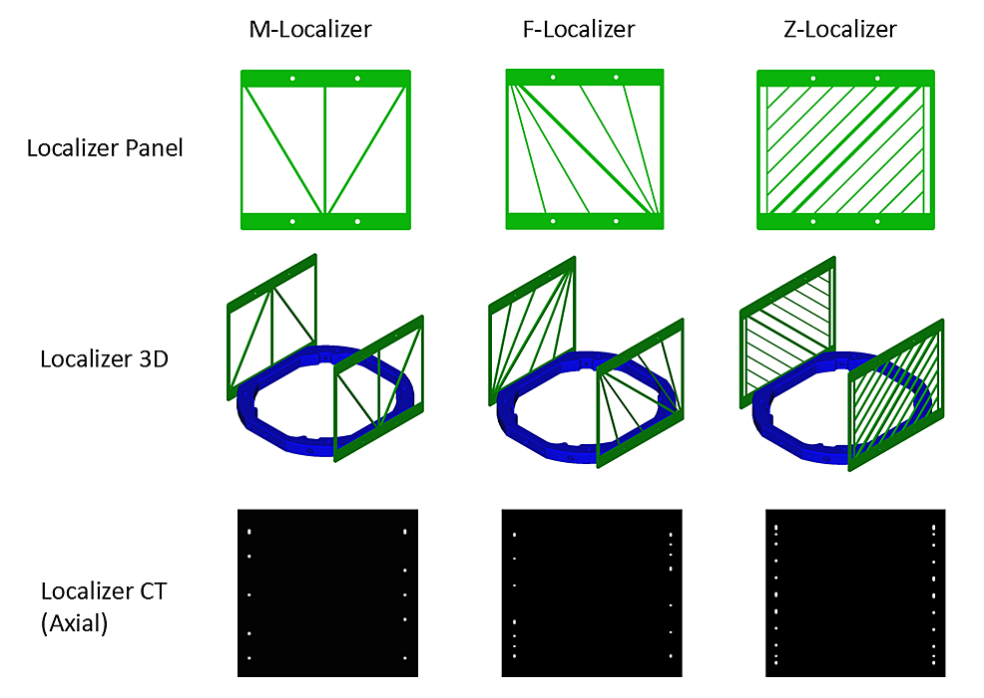
Three Novel Localizers: M, F, and Z. The top row of images depicts the individual localizer panels. The middle row presents a 3D view of each bipanel arranged in an antiparallel configuration. The bottom row illustrates axial computed tomography (CT) images created by the three bipanels.

Three new localizer panels were developed and simulated to better understand their utility for frame-based stereotactic localization. We have coined the names M-localizer, F-localizer, and Z-localizer (Figure 2). The M-localizer comprises an M-shaped set of fiducials with an additional central vertical bar. Thus, the M-localizer comprises three vertical bars and two diagonal bars per panel. The F-localizer comprises a primary diagonal bar, similar to the N-localizer, but with additional variable-angle diagonal bars. In total, the F-localizer comprises two vertical and five diagonal bars per panel. The Z-localizer, the most accurate and complicated panel system, comprises two vertical and 12 diagonal bars per panel. A bipanel arrangement doubles the number of bars for an axial image within the localizer volume for each of these panels. The Z-localizer comprises diagonal bars that all have a slope of 45 degrees relative to the vertical bars, and 10 of its 12 diagonal bars do not cross the entire stereotactic volume vertically.

Moreover, the Z-localizer has supporting vertical bars to which 10 diagonal bars abut; these supporting vertical bars are not intended for use in stereotactic calculations. All of these new localizer panels (M, F, and Z) are arranged in an "antiparallel" manner (i.e., one panel inverted compared to the opposite panel) that was found previously to minimize errors [3]. Each diagonal bar is bracketed by parallel, vertically oriented bars and can therefore exploit N-localizer mathematics to calculate the (x,y,z) coordinates of a point along a diagonal bar, given the (u,v) coordinates of 3 fiducials in a CT or MRI image [6]. Cross-sectional axial images were created and converted to Digital Imaging and Communications in Medicine (DICOM) images (Figure 2). Analysis of these images via techniques such as maximally stable extremal regions (MSER) predicts that the bars must be separated by approximately 2mm to prevent adjacent fiducials from merging. Merged fiducials cannot be used to calculate the (x,y,z) coordinates of a point along a diagonal bar because the (u,v) coordinates of the merged fiducials cannot be determined accurately and hence introduce a fiducial localization error (FLE) [7]. Merging can occur, for example, when a vertical bar abuts a diagonal bar, as will be discussed below relative to the Z-localizer.

Monte Carlo Simulation of Errors

**Figure 3:**
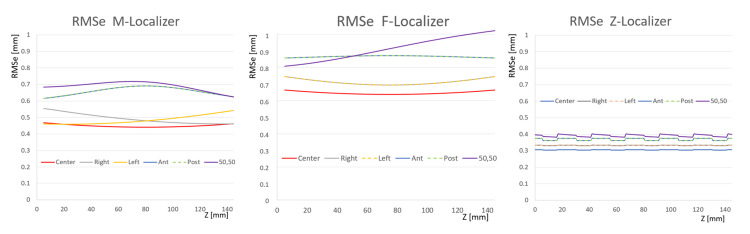
Plots of Root Mean Square error (RMSe) versus height (z) of each 2-panel (bipanel) system arranged in an antiparallel configuration. Monte Carlo (MC) simulations introduced millions of perturbations of $\pm$ 1mm random noise into the fiducial $\left ( u, v \right )$ coordinates. The resulting errors in calculated $\left ( x, y, z \right )$ target coordinates for targets having $\left ( u, v \right )$ coordinates expressed in units of millimeters at center $\left ( 0,0 \right )$; right $\left ( 50 ,0 \right )$; left $\left ( -50 ,0 \right )$; anterior $\left ( 0, 50 \right )$; posterior $\left ( 0, -50 \right )$; and anterolateral $\left ( 50,50 \right )$ are plotted for the M-localizer (left), F-localizer (middle), and Z-localizer (right). These errors demonstrate the Z-localizer to be the most accurate of the three systems.

Previously, Monte Carlo (MC) simulations were utilized to predict potential errors related to stereotactic localization [3]. These errors were reported as Root Mean Square Error (RMSE) and reflect millions of simulations for each system. After conceptualization of the three novel localizer bipanels, we implemented MC simulations to evaluate RMS-e. Using MC, we introduced random errors in the range of -1mm to +1mm into the 2D coordinates (u,v) of the fiducials in the image. We then analyzed how these errors propagated into the calculation for a target displaced from the centre of the image by 50mm anterior, posterior, lateral, and lateral. The Z-localizer had the least associated error throughout the volume, the M-localizer had the second least associated error, and the F-localizer had the most associated error (Figure 3). Note that the errors are generally on the order of the nominal size of one pixel or less for an MRI or CT scan (0.6-0.8mm).

## Discussion

Frame-based, image-guided stereotactic localization has been in use for longer than 40 years. The method creates fiducials in a stereotactic image so that they can be used to generate (x,y,z) coordinates that are translatable to surgery and thus yields a precise system for targeting regions of the brain. While extant systems generally comprise 3 or 4 N-localizer panels, the ergonomics of a 2-panel (bipanel) system that omits the anterior and posterior panels is attractive. However, using two N-localizer panels is insufficient for the stereotactic localization of a single image plane. Information from adjacent images correlated via 3D linear regression can provide sufficient data to compute stereotactic localization, but this technique can be fraught with inaccurate distortions causing error propagation [8]. Therefore, we have developed three novel panel systems that can be accommodated to a bipanel setup and thereby allow for proper stereotactic localization using a single image.

The novel bipanel systems give rise to an overdetermined set of equations that optimize the computations [3]. While the Z-localizer fared best in accuracy, this accuracy came at the cost of increased complexity. The Z-localizer comprises bars that do not span the entire imaging volume; thus, not all bars are visible and useable in a given axial image. For example, the two fiducials created by a vertical supporting bar and the abutting diagonal bar merge into a single fiducial near the abutment of the two bars, which can cause ambiguous identification of the fiducials. To eliminate ambiguity for the Z-localizer, we employed a strategy that uses two vertical bars and two diagonal bars from each panel that spans the entire volume. Therefore, for each image in which the bipanel localizer bars are visualized, four diagonal bars can accurately localize the image plane. After this initial plane localization, more diagonal bars can be added to improve localization accuracy. Most, but not all, of the diagonal bars may be added in this manner to improve accuracy. The choice of which specific diagonal bars to add involves filtering the diagonal fiducials by (1) examining the (u,v) coordinates of each diagonal fiducial relative to the (u,v) coordinates of the two vertical fiducials that they lie between and/or (2) fitting the (u,v) coordinates of the diagonal and vertical fiducials to a 2D regression line. The details of this filtering are complex and beyond the scope of the present article. Nevertheless, this complexity is rewarded with improved accuracy that approaches a four N-localizer system [3].

The M-localizer is an attractive bipanel system because it exhibits both simplicity and accuracy. In contrast to the bars of the Z-localizer, all bars of the M-localizer span the entire imaging volume, so interpretation of the fiducials is not susceptible to ambiguity; such ambiguity is the principal weakness of the Z-localizer. The additional central vertical bar permits calculation of the Pythagorean distances between the (u,v) coordinates of the fiducial created by this central vertical bar and the (u,v) coordinates of the fiducials created by the two outer vertical bars; if the 3 vertical fiducials have been correctly identified, the two distances should equal one another. A further test for correct identification of fiducials involves fitting the (u,v) coordinates of the diagonal and vertical fiducials for each panel to a separate 2D regression line. For each panel, all fiducials should closely fit the regression line. In addition, for imaging gantry tilts of 10 degrees or fewer, a secondary calculation similar to the previously published Sturm-Pastyr calculation can be included to improve accuracy [9, 10]. With or without this secondary calculation, the two diagonal bars per panel yield four diagonal bars, which together yield a system of overdetermined equations that improves accuracy. An interesting characteristic of the M-localizer is that a slight tilt in any direction may improve accuracy, as has been reported previously [10]. 

The F-localizer bipanel system is another simple system and is essentially an N-localizer with additional diagonal bars with variable slopes. These additional diagonal bars permit an overdetermined system of linear equations that improves accuracy. However, the steeper the slope of a given diagonal bar, the more error it introduces into the calculation [3]. The increased error associated with the steep diagonal bars of the F-localizer may be mitigated by weighting the contribution of a more steep bar to a lesser degree than the contribution of a less steep bar. Despite such mitigation, the F-localizer's increased sensitivity to noise compared to the sensitivities of the Z-localizer and M-localizer renders it less optimal than those localizers. As is also the case for the M-localizer, all bars of the F-localizer span the entire imaging volume, so interpretation of the fiducials is not susceptible to ambiguity. Furthermore, as is also the case for the Z-localizer and M-localizer, a test for correct identification of fiducials involves fitting the (u,v) coordinates of the diagonal and vertical fiducials for each panel to a separate 2D regression line.

**Figure 4:**
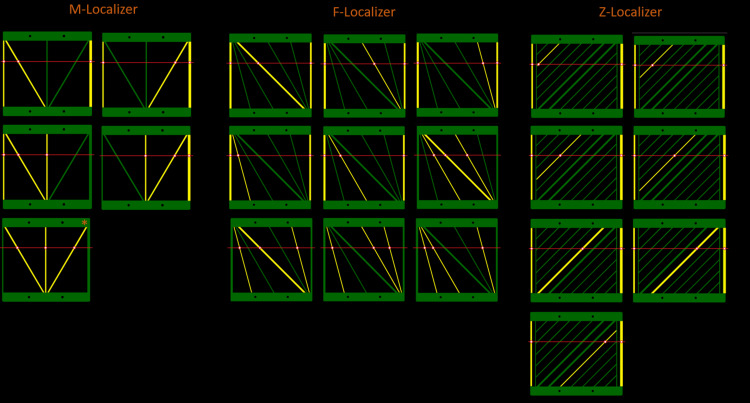
Localizer calculations. For the M-localizer, F-localizer, and Z-localizer, a minimum of three bars per bipanel system are required to calculate a 3D position within that image. For a given axial slice (red horizontal line), intersections with three bars (yellow lines) are utilized to calculate the position. Specifically, a diagonal bar that lies between two parallel bars is used to compute the 3D $\left ( x, y, z \right )$ coordinates of a point that lies on the diagonal bar. All of the calculations utilize N-localizer mathematics, with the exception of the last panel depicted for the M-localizer, which comprises two diagonal bars and one vertical bar and hence utilizes Sturm-Pastyr mathematics (*). A bipanel configuration for each localizer optimizes computation of the stereotactic matrix because each of the two panels provides more than three paired 3D $\left ( x, y, z \right )$ and 2D $\left ( u, v \right )$ coordinates. For example, each M-localizer, F-localizer and Z-localizer panel comprises respectively 5, 9, and 7 sets of three bars, where each set contains a diagonal bar bracketed by two parallel bars. Typically, the two parallel bars are vertical, but the F-localizer comprises 4 sets of bars wherein the parallel bars are diagonal instead of vertical.

Each bipanel localizer provides multiple sets of three bars to be used to calculate a 3D position (Figure 4). The majority of the calculations utilize standard N-localizer mathematics. In addition, the M-localizer provides the ability to use the Sturm-Pastyr calculation [9,10] for the central vertical rod, which we estimate to enhance the calculation when the gantry tilt is less than 10 degrees. In all cases, the bipanel setup optimizes the computation of the stereotactic matrix because there are more than three paired 3D (x,y,z) and 2D (u,v) coordinates.

An important consideration in all of these localizer systems is the concept of interpolation versus extrapolation. Prior publications analyzed the stereotactic triangle formed by the (u,v) coordinates of the diagonal fiducials created by a 3 N-localizer system [3,11,12]. It was found that surrounding a target with fiducials permits interpolation that minimizes error propagation for the target. Alternatively, when fiducials do not surround a target, then extrapolation is required. For example, a 2-panel N-localizer system, wherein each panel is affixed to one lateral side of a rectangular frame, permits interpolation except near the anterior and posterior edges of the image where extrapolation is required. This extrapolation degrades the accuracy of localization. In contrast, 3 and 4 N-localizer systems surrounding the patient require no extrapolation and yield the highest accuracy [3].

**Figure 5:**
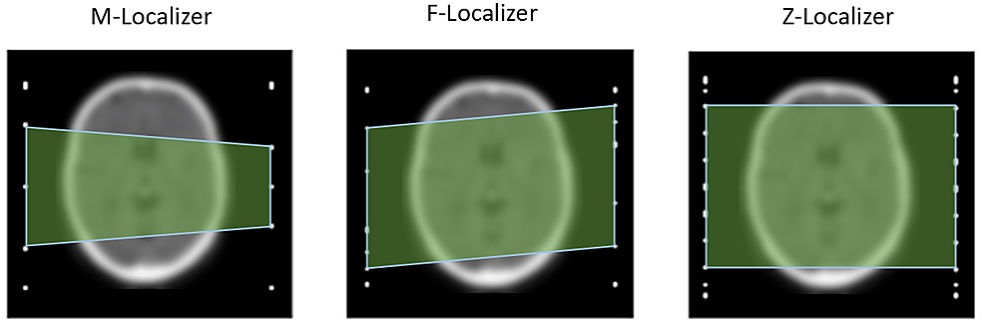
Representative quadrilateral coverage areas are depicted in light green for the M-localizer (left), F-localizer (center), and Z-localizer (right) using simulated axial images. The Z-localizer achieves the most coverage, the F-localizer achieves intermediate coverage, and the M-localizer achieves the least coverage but is more accurate than the F-localizer. The Z-localizer that achieves the most coverage is the most accurate of the three 2-panel (bipanel) localizer systems.

Notwithstanding the higher accuracies of 3 and 4 N-localizer systems surrounding the patient, the Z-localizer, M-localizer, and F-localizer fare well compared to these surrounding N-localizer systems. The accuracies of the Z-localizer, M-localizer, and F-localizer bipanel systems are high because each system creates a quadrilateral in the medical image that covers a reasonable fraction of the image (Figure 5). In general, the greater the area covered by the quadrilateral, the higher the accuracy [3]. However, although Figure 5 demonstrates that the quadrilateral created by the F-localizer subtends a larger area than the quadrilateral created by the M-localizer, the F-localizer is less accurate than the M-localizer. The reason for the paradoxically lower accuracy of the F-localizer is that its diagonal bars have a steeper slope on average than the diagonal bars of the M-localizer.

## Conclusions

Frame-based localization is accomplished using localizer systems, such as the N-localizer and the Sturm-Pastyr localizer. The highest localization accuracy is achieved by using 3 and 4 N-localizer systems that surround the patient. Ergonomic design and patient comfort have motivated introducing a 2 N-localizer system (bipanel) that fails to create sufficient fiducials in a single CT/MRI image to localize that image in a 3D stereotactic coordinate system. This article proposes several novel bipanel localizer systems wherein each panel comprises more than one diagonal rod; these localizer systems create sufficient fiducials to localize a single CT/MRI image. While all three of these novel bipanel systems appear to have high accuracy, as determined by our Monte Carlo simulations, the M-localizer achieves intermediate comparative accuracy while providing simplicity of fiducial interpretation. In contrast, the Z-localizer requires more complexity to interpret the fiducials but achieves accuracy that approaches the accuracy of a 4 N-localizer system.
